# Local Aphid Species Infestation on Invasive Weeds Affects Virus Infection of Nearest Crops Under Different Management Systems – A Preliminary Study

**DOI:** 10.3389/fpls.2020.00684

**Published:** 2020-06-25

**Authors:** Attila-Károly Szabó, Éva Várallyay, Emese Demian, Anna Hegyi, Zsuzsanna Nagyné Galbács, József Kiss, János Bálint, Hugh D. Loxdale, Adalbert Balog

**Affiliations:** ^1^Department of Horticulture, Faculty of Technical and Human Sciences, Sapientia Hungarian University of Transylvania, Târgu Mureş, Romania; ^2^Institute of Plant Protection, Faculty of Agricultural and Environmental Sciences, Szent István University, Gödöllő, Hungary; ^3^Molecular Plant Pathology Group, Department of Genomics, Agricultural Biotechnology Research Institute, Agricultural Research and Innovation Centre, Gödöllő, Hungary; ^4^School of Biosciences, Cardiff University, Cardiff, United Kingdom

**Keywords:** invasive weeds, virus vector, aphids, crops, cropping systems, small RNA, HTS

## Abstract

In the present study, we conducted field surveys to detect the population density of the most important invasive weed species and their associated virus vectoring aphids in crops grown under high input field (HIF) vs. low-input field (LIF) conditions, with and without fertilizers and pesticides. The most frequent invasive weed species were annual fleabane, *Erigeron annua* (L.), Canadian horseweed, *Erigeron canadensis* (L.) and Canadian goldenrod, *Solidago canadensis* (L.). These species were predominantly hosts of the aphids *Brachycaudus helichrysi* and *Aulacorthum solani* under both management systems. The 13% higher coverage of *E. annua* under LIF conditions resulted in a 30% higher *B. helichrysi* abundance and ∼85% higher *A. solani* abundance compared with HIF conditions. To reveal the incidence of virus infection in crop plants and invasive weeds, high-throughput sequencing of small RNAs was performed. Bioinformatics analysis combined with independent validation methods revealed the presence of six viruses, but with strikingly different patterns under LIF and HIF conditions. Their presence without symptoms in invasive weeds and crop plants supports the necessity of employing new approaches to those currently employed in invasive weed management. These findings also suggest that invasive weeds could serve as hosts for local aphid species and reservoirs for plant pathogenic viruses, both under low and high input management systems. In this light, as here demonstrated, viruses transmitted by local aphid species were found to differ between the management systems; hence, the importance of *B. helichrysi* and *A. solani* as virus vectors in particular clearly needs to be re-evaluated. Altogether, we accept that the present study is a pilot one and individual virus vectoring of aphids still needs to be directly tested. Even so, it represents one of the first contributions to this particular area, and thereby paves the way for further similar applied research in the future.

## Introduction

Invasive weeds represent a serious threat to world crop production as global trade expands and climatic conditions shift (Agrow, 2015; Usda Forest Service, 2015). In this light, it has recently been suggested that the losses to crop yield caused by invasive weeds and their infesting aphids (Hemiptera: Aphididae), may increase substantially (by around 25%) within the EU over the next few years (Usda Forest Service, 2015; EPPO, 2017). Such weeds are particularly important because they not only serve as hosts for several local sap-feeding, virus-vectoring insects, especially aphids, but also because they may act as significant reservoirs of pathogenic plant viruses (Frey et al., 2003; Zimmermann et al., 2015).

Previous studies from Central Europe have reported 435 alien weed species from 82 plant families in the past 25 years (Anastasiu and Negrean, 2005). Most of these species, for example annual fleabane, *Erigeron annua* (L.), Canadian horseweed, *Erigeron canadensis* (L.), and goldenrod, *Solidago canadensis* L., occur in all types of habitat and are considered to be the most important and economically-relevant invasive weeds within the agro-ecosystem (Anastasiu and Negrean, 2005). Although weed control strategies involve apparently useful approaches, including physical (e.g. mulching, tilling, burning) and chemical and other control (e.g. use of high quality seeds, crop rotation, herbicide application) (Chitsaz and Nelson, 1983; Rand and Louda, 2004; Uchino et al., 2012; Mabuza et al., 2013), the net areas covered by invasive weed species continues to increase in Central European regions (Tunaitienė et al., 2015; Pacanoski, 2017).

An important factor worthy of consideration in relation to this topic is the effect of these weed species on local sap-feeding pest insect populations, especially aphids. Another factor is that invasive weeds acting as virus reservoirs pose a serious threat via local aphid species in terms of virus distribution and transmission to crop plants. From this standpoint, several virus vectoring aphid species whose host range naturally includes local plant species, mainly from the same family e.g. Asteraceae, have become even more important, feeding and reproducing on invasive weeds (Bell, 1983; Popkin et al., 2017). Direct and indirect interactions between weeds and crop plants in close proximity can influence each other’s susceptibility (“associational susceptibility”) and affects their herbivore abundances (Barbosa et al., 2009). However, aspects on natural habitat diversity (i.e. diverse habitat surrounded by natural landscape mosaics) and how management systems (low vs. high chemical input) influence associational susceptibility or resistance have rarely been included in such analyses (Steffan-Dewenter et al., 2001).

In accordance with these various factors, the aim of the present study was: (1) to assess the population density of the most important invasive weeds under differently managed agricultural systems (high-input fertilizers and chemical pesticides (high-input fields, HIF) vs. no chemical management (low-input fields, LIF); (2) to identify and compare population densities of the most frequent aphid species on the most frequent invasive weeds; and (3) survey and identify plant viruses using high-throughput small RNA sequencing of invasive weeds and surrounding crops under different management regimes.

## Materials and Methods

### Study Area, Focal Weed and Aphid Species

Field surveys were conducted over a 2-year period during the crop growing seasons of 2015 and the 2016 in Central and Eastern Transylvania, Romania. The methods used have also been described by Szabó et al. (2019). Assessments were run under two different input management regimes (low and high), carefully selected to take into consideration similar geographic and climate regions (see also / Supplementary Figure S1).

#### Low-Input, Traditionally Managed Fields (LIF)

This area belongs to a traditionally managed field (low-input) of the Old Saxon cultural region covering an area of ∼ 7,440 km^2^ at an altitude ranging from 230 to 800 m above sea level (a.s.l.) and characterized by a landscape mosaic of different land-cover types (28% forest, 24% pasture, and 37% arable land, mostly maize, potato, and alfalfa). The farming practices are predominantly small scale subsistence farming, with no chemical inputs. One consequence of this kind of land use is the exceptional biodiversity and natural value of the farming landscape (Akeroyd and Page, 2011). However, being not particularly viable economically, the abandonment of croplands in this region is frequent, resulting in the establishment and high abundance of invasive weeds (Zimmermann et al., 2015).

#### High-Input, Conventionally Managed Fields (HIF)

This study region contrasts the previously described region by growing large monocultures and farming landscapes with low levels of natural vegetation. The area of about 5,500 km^2^ at altitudes between 220 and 750 m a.s.l. has been, and continues to be, intensively treated with synthetic fertilizers and pesticides, major crops including maize, potato, and alfalfa (Supplementary Table S1). The studied fields inside the described region were situated at the same altitudinal range of about 250 m a.s.l. and under comparable bioclimatic conditions. The distance between the studied areas was roughly 200 km, with no direct connections (main roads and railways) between regions.

Previous assessments confirmed that three weed and two native aphid species are frequent in both regions, so these particular plant hosts and their infesting aphids were studied and sampled. The most important weed species found were the annual fleabane, *Stenactis* (=*Erigeron*) *annua* (L.), Canadian horseweed, *Erigeron* (=*Conyza*) *canadensis* (L.), and Canadian goldenrod, *Solidago canadensis* (L.). These species are known to grow in a diverse range of habitats and are considered important weeds in Europe, causing substantial crop losses following colonisation of new areas (Anastasiu and Negrean, 2005). *Erigeron annua* is often a dominant species within invasive weed communities, and has been reported from almost all European countries, its expansion increasing over recent years (Edwards et al., 2006; Tunaitienė et al., 2015; Pacanoski, 2017). *Erigeron canadensis* is an annual plant native throughout most of North and Central America. It is also widely naturalized in Eurasia (Nandula et al., 2006; Shah et al., 2014; Bajwa et al., 2016). *Solidago canadensis* is a perennial weed native to north-eastern and north-central America, but has established as an invasive throughout Europe (Abhilasha et al., 2008; Fenesi et al., 2015).

The two native aphid pests species examined in this study were the highly polyphagous leaf-curling plum aphid, *Brachycaudus helichrysi* (Kaltenbach) and the similarly polyphagous foxglove aphid, *Aulacorthum solani* (Kaltenbach) (Blackman and Eastop, 2000; Blackman, 2010). These are particularly important species, not only because of their wide host range, but also because of their diverse virus transmission. The host plant range of *B. helichrysi* includes members of the Asteraceae, e.g. Chrysanthemum, species of *Prunus* and also species of *Solanum*, *Fragaria*, *Trifolium, Medicago*, *Citrus* and maize, *Zea mays* (Tatchell et al., 1983; Powell et al., 1992; Isac et al., 1998; Popkin et al., 2017). Viruses transmitted by this aphid include Plum Pox virus, Potato virus Y and Beet mild yellowing virus (Isac et al., 1998). Host plants of *A. solani* include several crop plants including tomato, peppers, tobacco, celery, carrots, tulip bulbs, cucurbits, and legumes (Tatchell et al., 1983; Jandricic et al., 2014). The most important viruses transmitted by it are Potato viruses A, Y and X and Potato leaf roll virus, Cucumber mosaic virus, Soybean dwarf virus, Bean yellow mosaic virus and Turnip yellows virus (Jandricic et al., 2010, 2014).

### Assessment of Invasive Weeds and Associated Aphids

Firstly, we selected two blocks of land approximately 3 km long and 1 km wide inside each management system (regions); these blocks were located 10 km distant from each other in LIF and in 15 km distant in HIF. Inside each block, we further established two transects (at least 1 km apart) of 10 m long × 1 m wide at an approximately equal distance (between 1 and 2 metre) to three major crops (maize, potato, and alfalfa) dominating (as crop) more than 95% of both management systems at field crop margins. In this way, each transect was surrounded by at least 8–10 ha of high-input, and at least 0.5–3 ha of low-input agricultural crops, including around weed populations dominated by maize, alfalfa and potato fields. The transect selections were made at the same period each year (between 5 and 8 May) and when all crop plants from both regions were at the same vegetation stage. The same transect and the same crop plant combinations were followed the next year. Each transect was carefully measured and located using GPS.

Secondly inside each transect, 10 × 1 m^2^ quadrats were placed. Each of these was further subdivided into 10 × 10 cm sub-quadrats (100 sub-quadrats inside each quadrat). Inside each of these sub-quadrats all plants (native and invasive) were counted and their coverage estimated (Andújar et al., 2010).

Thirdly, ten individual invasive weed plants from each sub-quadrat were randomly collected by hand and placed in plastic bags. The number of invasive plants collected for each species from each sub-quadrat mirrored the coverage of the species within the quadrat. We decided, subjectively, that at least eight plants be collected when the coverage of a given species in a sub-quadrat was at least 80% and comprised up to two plants if the coverage of the species was up to 20%. We decided upon these percentage coverage thresholds because in each quadrat there was one highly dominant invasive plant species (its coverage having at least 80%) and one species which had a coverage between 15 and 20%. Therefore, from each sub-quadrat, out of the 10 plants sampled, at least eight belonged to the dominant species and one or two to the second most dominant species.

Because plants contained aphid colonies, and the exact number of individual aphids was important, all plastic bags were labelled and kept at low temperature (∼0–4°C in a cool box), then returned to the laboratory, whereupon all samples were stored at −20°C, with aphids later counted and species identified (Blackman and Eastop, 2000; Blackman, 2010). In total, 100 plant samples were collected per transect and management system (400 samples per management system per collection data, and a total 1600 samples/year). The same methods of using quadrat dimensioning were followed in crop plants around each transect, i.e. equal distance (between 1 and 2 metre) to major crops (maize, potato, and alfalfa) dominating sites, except with regard to counting percentage plant coverages; here only plant material (the same number of samples as from weeds) were collected and stored for aphid assessment and virus detection.

Assessment began at the end of May and was repeated fortnightly five times during the summer until the end of the weed growing season, whereupon no more aphids were found. The whole procedure was repeated in the following year using the same collection methods within the same transects. All aphids were carefully counted under laboratory conditions, and the plant materials were used for virus identification employing metagenomic high-throughput sequencing (HTS) of small RNAs as an unbiased method, able to detect all viruses present in the sample (Roossinck et al., 2015).

### Virus Detection From Invasive Weeds and Crop Plants

Leaf samples on which aphids had been found and counted were used for viral analyses from both weeds and crop plants (alfalfa, maize, and potato) and both management systems, with small RNA analyses performed in 2017. RNA was extracted using a phenol-chloroform method (White and Kaper, 1989). Briefly, frozen plant material was homogenized in an ice-cold mortar, suspended in 650 μl of extraction buffer (100 mM glycine, pH 9.0, 100 mM NaCl, 10 mM EDTA, 2% SDS and 1% sodium lauroylsarcosine) and mixed with an equal volume of water saturated phenol, and centrifuged for 5 min. The aqueous phase was treated with equal volumes of phenol, chloroform, and isoamyl-alcohol (25:24:1), and after subsequent treatment with chloroform: isoamyl-alcohol (24:1), was precipitated with 99.8% ethanol and then re-suspended in sterile water.

For small RNA HTS, small RNA was isolated from polyacrylamide gels involving RNA pools which were prepared by mixing equal amounts of RNA originating from different individuals, collection times (in the case of weeds) and from different species in the case of crops (Supplementary Table S2). This pooling strategy allowed detection of any virus present in any of the sampled individual plants at any time during the survey. As the crops investigated belonged to different families, and as such hosted very different viruses, we investigated their viral patterning as a collective pool. In contrast, the invasive weeds were all members of the Asteraceae; hence virus diagnostics were here performed separately for each species concerned.

These pools were used for small RNA library preparation (six libraries in total) using Truseq Small RNA Library Preparation Kit (Illumina, United States) and our modified protocol (Czotter et al., 2018). Samples were sequenced using HiScan2000 by UD Genomed (Debrecen, Hungary) 50 bp, single end. Fastq files of the sequenced libraries were deposited to the GEO and can be accessed through series accession number GSE132755.

### Virus Diagnostics by RT-PCR and Northern Blot

Pooled RNA extracts or RNA extracts prepared from the individual crops were used as templates for cDNA synthesis by a RevertAid First Strand cDNA Synthesis Kit (Thermo Fisher Scientific, United States) with random primers according to the manufacturer’s instructions. The generated cDNA was then used for PCRs (primers amplifying viral parts are provided in Supplementary Table S6) performed with Phire Hot Start II DNA Polymerase (Thermo Fisher Scientific). Results were analysed using 1.2% agarose gel-electrophoresis, and PCR products were subsequently Sanger sequenced to prove them to be virus specific.

For Northern blot analysis, 4 μg of total RNA (the same pooled samples used for small RNA library preparation) were separated on a formaldehyde-containing 1.2% agarose gel and blotted to a Hybond-N membrane. Radioactively labelled random DNA probes were generated from cloned, purified PVX PCR product with a Decalabel DNA Labeling Kit (Thermo Scientific). Northern blots were hybridized with this probe in Church buffer (1% BSA (bovine serum albumin), 1 mM EDTA, 0.25 M Na_2_HPO_4_, 7% SDS, pH 7.2) at 65°C, washed according to the manufacturer’s instructions and exposed to X-ray film.

### Data Analysis

For weed data, the mean coverage per 1 m^2^ sub-quadrat was determined by averaging the plant values from each 10 × 10 cm plot. Next, the inter-annual differences in coverage were tested using multivariate analysis of variance (MANOVA) and mean coverage values obtained for one 1 m^2^ quadrat (40 data/field type/collection dates) were considered. No significant difference in weed coverage were detected between years (*P* = 0.12). Therefore, data from the 2 years, collected on the same dates, were combined for the analyses. The weed frequency data were tested using Poisson-distributed errors residuals for normality of errors (Kolmogorov-Smirnov test) and for equality of variance (Levene’s test). Because residuals did not meet the assumption of normality, we used the non-parametric Kruskal-Wallis- and Mann-Whitney *U* test to compare variables. Weed species and management systems (HIF vs. LIF) were used as fixed factors and the average weed coverage in 1 m^2^ sub-transect as random factor.

We next determined how the cropping system differentially affected associational susceptibility to the two aphid species, *B. helichrysi* and *A. solani*. General linear modelling was used with mean aphid abundance on *E. annua, E. canadensis* and *S. canadensis* as response variable. Initial analyses indicated no difference between study years and aphid abundance averaged across study years (*P* < 0.23). The model included cropping system type (HIF vs. LIF), aphid species (*B. helichrysi* and *A. solani*), and their interaction as explanatory variables. Because aphid abundance is a discrete variable, Poisson-distributed errors were assessed. Aphid abundances on *E. annua* was normally distributed, so factorial ANOVA was used, followed by Tukey testing. Aphid abundance on *E. canadensis* and *S. canadensis* did not meet the assumption of normality, hence the Kruskal-Wallis test was used, followed by the Mann-Whitney *U* test.

Significant (*P* < 0.05) interactive effects (cropping system type × species) suggested that the effect of cropping system depended on aphid species. Aphid density analyses on crop plants were made considering only the abundance of *B. helichrysi.* As the data was normally distributed, ANOVA was used, followed by Tukey testing to compare abundances between treatments and crop plants. The density of *A. solani* was only high in potato, but there were no significant differences between treatments (*T*-test *P* = 0.78); therefore no other analyses at this species abundance on crops were made. All analyses were performed using R version 3.0.1 (R Core Team, 2013). Only a small number of other aphid species (e.g. *Macrosiphum* spp.) were detected, and we did not include them in the analyses.

Principal Components Analysis (PCA) was used to identify the proportion of variation in each PCA axis (Aphids density and treatments) explained by each virus distribution. We then used the average count of each virus reads numbers detected and log10 transformed from each weed and crop plant sample grouping as response variables, and used aphid abundance as component 1 (PCA axis1) and treatment (LIF vs. HIF) as component 2 (PCA axis 2) scores for each virus reads as independent variables. RNA and DNA viruses were analysed separately, and the only one insect virus detected (Helicoverpa zea nudivirus 2, HzNV-2) was not considered in our analyses. PCA covariance analyses were run using Community Analysis Package 4 (Pisces Conservation Ltd).

Virus diagnosis was determined by small RNA HTS. For bioinformatics analysis of the HTS results, we used CLC Genomic Workbench. Briefly: for trimming, quality control and QC reports, embedded protocols in CLC Genomic Workbench were employed. For virus diagnostics, we followed two strategies and used CLC Genomic Workbench: we built longer contigs from the non-redundant reads using assembler of CLC (*de novo* assembly) and compared the resultant contigs using BLAST to the NCBI Reference Genomes of plant viruses downloaded from GenBank. In parallel, we directly mapped contigs to Reference Genomes of those viruses which were represented at least with one contig in any of the libraries mapping to the reference tool of CLC Genomic workbench. Virus presence was recorded if at least two parameters were reached, i.e. virus specific contig was present and/or normalized redundant virus specific read count was >200, and/or coverage of the virus genome was >60%.

## Results

### Dominant Invasive Weed Species and Their Variations Between Management Systems

**FIGURE 1:**
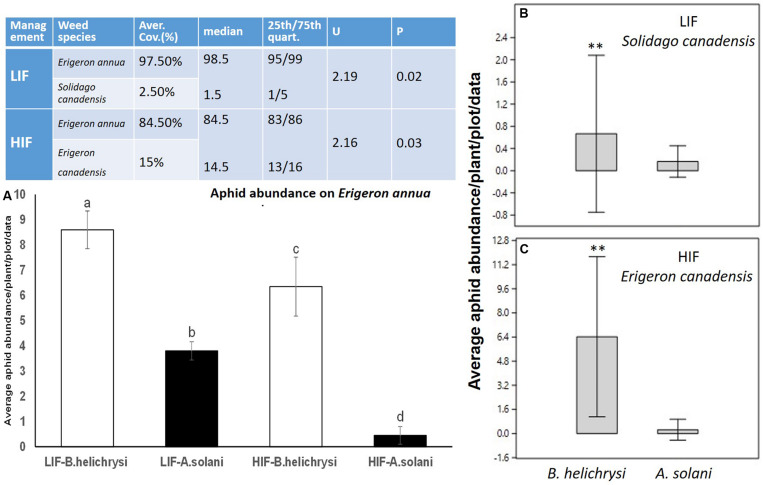
The coverage of the most frequent weed species between LIF and HIF regimes (Table) and the abundance of the most frequent aphid species between treatments **(A)** and weed plants **(B,C)**. Weed data were compared using non-parametric Kruskal-Wallis- and Mann-Whitney *U* tests. Aphid abundance on *E. annua* was normally distributed, so factorial ANOVA was used, followed by Tukey testing. Aphid abundance on *E. canadensis* and *S. canadensis* did not meet the assumption of normality, hence the Kruskal-Wallis test was used, followed by the Mann-Whitney *U* test. Significance level = *P* < 0.05. ^∗∗^*P* < 0.01. Different letters (a–b) means statistical significant difference.

Three weed species, all considered invasive, were dominant during the 2 years field assessment. *Erigeron annua* was the most frequent, dominating both LIF (97.5%) and HIF treatment regimes (84.5%). Two other species were present at lower densities. *S. canadensis* was only present in LIF, with a coverage of 2.5%. No other invasive weeds were assessed under this management system. *E. canadensis* was only present under HIF with a coverage of 15%. Other weed species, mostly amaranth, *Amaranthus* spp. in HIF regimes with an average coverage of 0.5%, were observed at the end of the growing period of the above mentioned weed species. Dominance of *E. annua* was significant under both management systems (Figure 1).

### Aphids and Their Abundances on Invasive Weeds and Crop Plants

**FIGURE 2:**
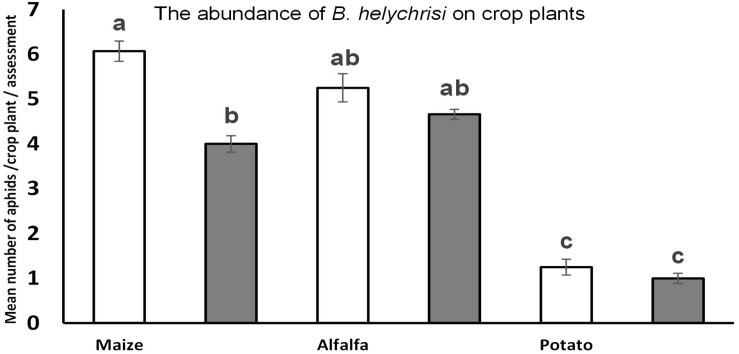
The abundance of *B. helychrisi* on crop plants under different treatments (HIF vs. LIF). ANOVA was used followed by Tukey testing to compare variables. Significance level = *P* < 0.05. Error bars represent standard errors. Different letters (a–b) means statistical significant difference.

Two native aphid species were detected at high density on all three dominant invasive weeds. The most frequent was *B. helichrysi*, which infested the most frequent weed, *E. annua*, under both management regimes (LIF-*B. helichrysi* and LIF-*A. solani F*_1__–__40_ = 6.4, *P* < 0.001; LIF-*B. helichrysi* and HIF-*A. solani F*_1__–__40_ = 8.1, *P* < 0.001) (Figure 1A). The next most abundant species was *A. solani*, also present on *E. annua* plants under both management systems; its density was significantly higher under LIF compared with HIF (*F*_1__–__40_ = 8, *P* < 0.001) (Figure 1A). A higher density of *B. helichrysi* was observed on *S. canadensis* under LIF (*U*_1__–__40_ = 3.4, *P* < 0.01) (Figure 1B). Furthermore, the dominance of *B. helichrysi* on *E. canadensis* was observed under HIF conditions (*U*_1__–__40_ = 3.1, *P* < 0.01) (Figure 1C). A very low number of other aphid species were observed, i.e. about 12 individuals of *Macrosiphum* spp. were collected on *S. canadensis*. The abundance of *B. helichrysi* was higher in maize under LIF compared with HIF (*F*_1__–__40_ = 4.5, *P* < 0.01). No other differences between treatments were observed in alfalfa (*F*_1__–__40_ = 0.5, *P* < 0.89) and potato (*F*_1__–__40_ = 0.2, *P* < 0.91) (Figure 2).

### Plant Viruses in Invasive Weeds and Crop Plants

Sequencing of the small RNA libraries resulted in 9.4–21 million raw reads (Supplementary Table S3). After trimming and quality control we obtained 9.2–20.4 million reads, which represented 2,019,811–7,192,941 individual sequences. Using overlapping stretches of these reads, we were able to construct *de novo* 3,553–19,038 longer contigs (Supplementary Table S3). Virus derived contigs were annotated following BLAST searches. Thereafter, we made direct mapping of the sequenced trimmed reads to each viral reference genome, which gave at least one hit according to contig annotation and counting all redundant and non-redundant reads derived from a given virus. To compare the number of reads in different libraries, normalized redundant reads (read/million read) were calculated. According to this approach the presence of 42 different viruses were detected.

Annotation can lead to false positive results in familiar viruses (Massart et al., 2019). Moreover, as we used pools for small RNA HTS, RNA from the non-infected plants could dilute the sample for virus specific reads – for example, a high, normalized, redundant read count could be counted even without the presence of the virus specific contig. Hence, the presence of the virus was counted *only* when the presence of the contig(s) coincided with a relatively high (>200) normalized redundant read and >>60% coverage of the genome.

**FIGURE 3:**
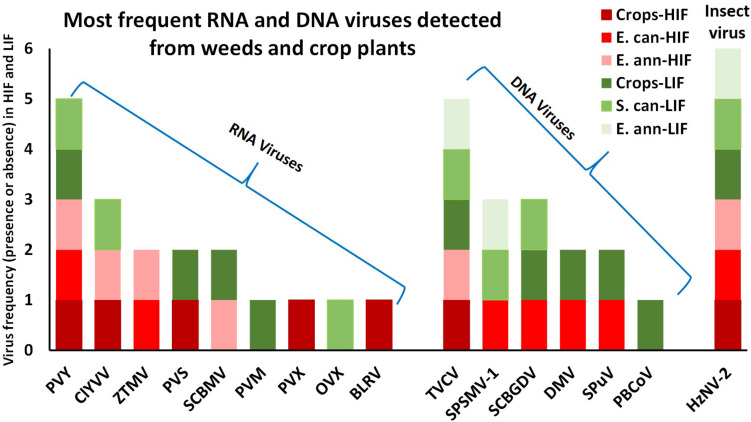
The presence of RNA and DNA viruses between crops and invasive weeds under low and high input field regimes. Red colours represents HIF, green colours represents LIF. E. ann - *Erigeron annua*, C. can - *Conyza canadensis*, S. can - *Solidago canadensis*. PVY, Potato virus Y; ClYVV, Clover yellow vein virus; ZTMV, Zucchini tiger mosaic virus; PVS, Potato virus S; SCBMV, Squash chlorotic leaf spot virus; PVM, Potato virus M; PVX, Potato virus X; OVX, Opuntia virus X; BLRV, Bean leafroll virus; TVCV, Tobacco vein clearing virus; SPSMV-1, Sweet potato symptomless mastrevirus 1; SCBGDV, Sugarcane bacilliform Guadeloupe D virus; DMV, Dahlia mosaic virus; SPuV, Soybean mild mottle pararetrovirus; PBCoV, Pineapple bacilliform comosus virus; HzNV-2,- Helicoverpa zea nudivirus 2.

Following this revised analytical approach, the number of viruses detected dropped to 16, differentiated as nine RNA and seven DNA viruses (Table 1, Figure 3, and Supplementary Table S4). The distribution of both RNA and DNA viruses varied greatly between crops and invasive weeds, both under LIF and HIF regimes. The most widespread virus detected was the insect baculovirus HzNV-2, which may have originated from larvae of the highly polyphagous corn earworm moth, *Helicoverpa zea* (Lepidoptera: Noctuidae) feeding in the fields sampled. Of plant viruses, PVY and TVCV were the most widespread, only lacking from library 6_SS and 2_TB, respectively. Crops grown under LIF contained nine viruses, while under HIF, only six were found. PVX and BLRV were present only in crops under HIF, while PVM and PBCoV were only present in LIF crops (Table 1, Figure 3, and Supplementary Table S4).

**TABLE 1:** The list of the 16 viruses detected in invasive weeds and associated crop plants using mild parameters in the bioinformatics analysis.

	Virus name		Genome	Host range	Vectors described until now
PVY	Potato virus Y	Potyvirus	RNA	infects at least 60 plant spp., mostly in the Solanaceae	Transmitted in the non-persistent manner by several aphid spp. *Myzus persicae* is probably the most efficient vector; others are *Myzus ornatus*, *Macrosiphum euphorbiae*, *Aulacorthum circumflexum* (= *Neomyzus circumflexus*), *Aphis nasturtii*, *Aphis gossypii* and *Brachycaudus helichrysi*
ClYVV	Clover yellow vein virus	Potyvirus	RNA	25 species in 6 plant families	Transmitted in the non-persistent manner by the aphids *Myzus persicae*, *Acyrthosiphon pisum*, *Aulacorthum solani* and *Macrosiphum euphorbiae*, but not by *Aphis fabae*
ZTMV	Zucchini tiger mosaic virus	Potyvirus	RNA	Cucurbitaceae, ucchini squash (Cucurbita pepo)	Aphids
PVS	Potato virus S	Carlavirus	RNA	Susceptible species belong mainly to the Solanaceae	Transmissible in the non-persistent manner by the aphid *Myzus persicae*, by the aphid *Myzus persicae*; less efficiently by *Aphis frangulae*, *A. nasturtii*, and *Macrosiphum euphorbiae*
PVM	Potato virus M	Carlavirus	RNA	Susceptible species belong mainly to the families Solanaceae and Chenopodiaceae	Isolates may differ in their transmissibility by *Myzus persicae*
PVX	Potato virus X	Potexvirus	RNA	The host range is mostly limited to the Solanaceae	Transmission is reported by the grasshoppers *Melanoplus differentialis* and *Tettigonia viridissima*, Transmission has also been reported by the fungus *Synchytrium endobioticum*
OVX	Opuntia virus X	Potexvirus	RNA	Opuntia, Cactaceae, Eukaryota; Viridiplantae; Streptophyta, Streptophytina, Caryophyllales, Chenopodiaceae; Chenopodioideae, Atripliceae, Chenopodium	No known insect vector
BLRV	Bean leafroll virus	Luteovirus	RNA	65 species of Vicia, Pisum, Medicago, Trifolium, Lathyrus and Trigonella	The principal vector of bean leaf roll virus is the pea aphid, *Acyrthosiphon pisum*. *Myzus persicae* is a less efficient vector
SCBMV	Squash chlorotic leaf spot virus	Picornavirales	RNA	Cucurbit	Transmitted mechanically and by two whitefly species, but not by aphids
TVCV	Tobacco vein clearing virus	Solendovirus	DNA	Eukaryota; Viridiplantae; Streptophyta; Streptophytina; Solanales; Solanaceae; Nicotianoideae; Nicotianeae	No known insect vector
SPSMV-1	Sweet potato symptomless mastrevirus 1	Mastrevirus	DNA	Sweet potato	Leafhopper transmission
DMV	Dahlia mosaic virus	Caulimovirus	DNA	Natural infection found only in Dahlia species, but the virus can infect 11 other members of the Compositae, and 13 species in the Solanaceae, Chenopodiaceae and Amaranthaceae	Transmissible by 13 aphid species, notably *Aphis fabae, Myzus persicae* and *Macrosiphum euphorbiae*
SPuV	Soybean mild mottle pararetrovirus	Caulimovirus	DNA	Soybean	No known insect vector
PBCoV	Pineapple bacilliform comosus virus	Caulimovirus	DNA	Pineapple	Mealybugs
SCBGDV	Sugarcane bacilliform Guadeloupe D virus	Caulimovirus	DNA	Sugarcane	Mealybug insects species
HzNV-2	*Helicoverpa zea* nudivirus 2	Baculovirus	DNA	Insects, Lepidoptera: Noctuidae, *Helicoverpa zea*	

The presence of potato infecting viruses were also investigated with independent methods, RT-PCR and Northern blot. Under HIF conditions, only PVX was detected, while under LIF, PVY, PVS, PVM were validated (Supplementary Table S5 and Supplementary Figure S2). Although we obtained many small RNA reads mapped to PVY and PVS, we could not validate their presence under HIF conditions (Supplementary Figure S2). It is possible that the antiviral silencing was very active in these circumstances, which explains why we detected the small RNAs, but not the entire viruses, the titre of which had declined. However, it is clear from the RT-PCR and Northern blot results that while viruses vectored by aphids (PVY, PVS, and PVM) are present under LIF conditions, PVX, which is mainly mechanically transmitted, is not, and instead was only detected in HIF regimes.

Several viruses detected during the present study have not been previously reported or have been rarely mentioned by the European and Mediterranean Plant Protection Organization, EPPO (Supplementary Table S6). In the case of the Opuntia virus X, its insect vector has not yet been described. No reports by EPPO concerning Tobacco vein clearing virus from Europe exist, whereas we detected this virus at high frequency in both crops and weeds under LIF and HIF regimes (Table 1 and Figure 3). This pararetrovirus was first described from a hybrid form of tobacco, *Nicotiana edwardsonii* (Lockhart et al., 2000), and found to be highly integrated into the host genome. There is still no additional TVCV deposited into GenBank. We made an effort in this respect and tried to amplify the virus in our samples. We obtained some product of the expected size, but subsequent Sanger sequence analyses showed that this arose from the host genome. We believe that such detection is a false positive and that we apparently had hits because some of the plant genomes host this type of retroviral element. The Sweet potato symptomless mastrevirus 1 is also absent from any EPPO alert lists, whereas we detected it in invasive weeds, but not in crops. The Pineapple bacilliform comosus virus has been reported from tropical areas, while we detected its presence at high read numbers in crops under LIF. The Sugarcane bacilliform Guadeloupe D virus, reported as frequent in the tropics, was detected in crops under LIF, but also in weeds, i.e. *E. canadensis* under HIF and *S. canadensis* under LIF. The Lepidoptera-infecting Helicoverpa zea nudivirus 2 has not been officially reported from Europe, and hence is not present in the EPPO listings. Its frequency was the highest in all crops and weeds under all management systems (Table 1 and Figure 3).

**FIGURE 4:**
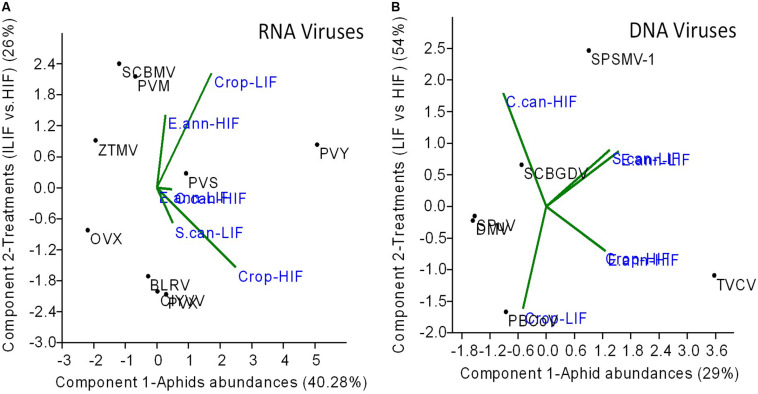
The effect of treatments (HIF vs. LIF and aphid *B. helichrysi* and *A. solani*) abundance on RNA **(A)** and DNA **(B)** virus distributions between crop plants and weeds. The only insect virus detected (HzNV-2) was not considered in the analyses. Principal Components Analysis (PCA) was used and the average count of each virus read from each weed and crop plant sample grouping were treated as response variables, and aphids abundance as component 1 (PCA axis1) and treatment (LIF vs. HIF) as component 2 (PCA axis 2) scores, related to each virus read as independent variables. RNA and DNA viruses were separately analysed.

Using PCA to identify the proportion of variation in terms of aphid density and treatments explaining RNA and DNA virus distribution, we observed that aphid abundance was the most important factor governing RNA virus distribution (40%), whilst treatments (LIF vs. HIF) had only a smaller effect (26%) (Figure 4A). In contrast, DNA virus distribution was mostly determined by treatment (54%) rather than aphid distributions (29%) (Figure 4B).

## Discussion

According to our present findings, it is clear that associational susceptibility exists between the most frequent invasive weed and crop plants under different crop management (LIF vs. HIF) regimes. As determined experimentally, a 13% higher coverage difference of *E. annua* in LIF further resulted in a significantly higher *B. helichrysi* abundance (about 30% more). The same trend was observed for *A. solani*, where a 13% higher coverage of *E. annua* resulted in an increase of about 85% for this aphid under LIF compared to HIF regimes (Figure 1). In our previous experiments testing colonization abilities of these particular aphids (Szabó et al., 2019), it was also shown that *E. annua* and *E. canadensis* are suitable weed hosts for both species but especially for *B*. *helichrysi.* Significant colonization of *B*. *helichrysi* from both weeds toward the crop plants maize, alfalfa, and potato was detected (Szabó et al., 2019). Therefore, virus transmission from invasive weeds to crop plants by these aphids is highly probable.

Several factors appear to influence associational susceptibility between invasive weeds and crop plants. The likelihood of detection of neighbouring plants, and the factors that can directly affect the survival of local aphid populations on these plants, seemingly include natural habitat diversity as well as lack of any chemical usage under the LIF regime. The short distance between weed populations and crop plants (here alfalfa, maize, and potato) and the high habitat diversity apparently may influence associational susceptibility of crop plants, and the presence of the two aphid species, especially *B*. *helichrysi*, can be explained in all three crop plants (Figure 2).

In addition, aphid abundance and the management systems used may directly influence plant virus distribution between weeds and crop plants, while RNA virus distribution (mostly aphidophag viruses transmitted through stylet-borne mechanisms) is probably more influenced by aphid density *per se*, and DNA virus distribution (transmitted less by aphids, probably more by mechanical means) between weeds and crops by contrast predominantly influenced by the management system involved (Figures 4A,B). While we made substantial efforts to extract and detect viruses from aphid stylets collected from weeds and crop plants during assessment and thereby confirm direct transmission, we were unsuccessful. This is undoubtedly due to two principal reasons: (1) The technique used may be fundamentally unsuited for virus detection from aphid stylets or (2) it may simply be that the titre of virus within the aphid stylets is very low, whereas in the plant itself, it is very much amplified, allowing detection. The previous colonization experiment (Szabó et al., 2019) clearly demonstrated that with both aphid species here studied, virus transmission definitely occurred during the insects’ movement from weeds to crop plants and the colonization of these.

Besides aphid-transmitted viruses, another important group of plant pathogenic viruses comprises those transmitted mechanically or indeed, by unknown means. In the case of PVX virus, as here presently found only in HIF regimes, if the tubers are infected, cultivators can readily disseminate it within and between potato crops. With PVM, it is usually present in herbaceous Solanaceous plants within the surrounding flora. We didn’t detect it in any of the Composite weeds sampled and tested, probably because they cannot host it. However, it was only present in the LIF regimes, suggesting that it could be transmitted by the sap sucking herbivorous insects like aphids feeding on these weeds. The same could happen with BLRV and PBCoV: since the Composite weeds do not host them, we hence failed to detect them in the libraries.

DNA viruses were detected in the crops within the LIF regimes, and contrastingly were present in weeds in both HIF and LIF, but here revealed a different pattern of infection (Supplementary Tables S2, S3 and Figure 3). This result is likely a consequence of the fact that weeds belonging to the family Compositae can host DNA viruses, which cannot be hosted by the crops investigated, such that the pattern of infection is further altered, a possibility that we naturally have had to take into consideration.

Lastly, several of the viruses detected in our study have not previously been reported from Europe, i.e. Opuntia virus X, Tobacco vein clearing virus, Sweet potato symptomless mastrevirus 1, Pineapple bacilliform comosus virus, Sugarcane bacilliform Guadeloupe D virus and the Helicoverpa zea nudivirus 2. Although our detection of these viruses by small RNA HTS seems credible, further studies involving a different approach (e.g. RT-PCR) need to be performed in order to add support to these findings. What is certain is that the presence of these viruses in both invasive weeds and crop plants without the production of pathological symptoms in the former raises the clear necessity of acknowledging the potential and indeed likely presence of such pathogenic disease reservoirs during invasive weed management scenarios.

## Conclusion

Overall, we conclude from these findings that even if low-input management farming systems are widely studied (e.g. Akeroyd and Page, 2011; Fischer et al., 2012; Mikulcak et al., 2013) and are supposedly low-cost, effective systems (i.e. no or low management costs) with high biodiversity and cultural values (Hartel et al., 2013), the abandonment or absence of management may cause serious problems. This is mainly due to the likelihood of increased virus vectoring aphid densities, including of hitherto unknown or known but unrecorded viruses, which further affect local cultivated plants, and may in addition influence local wild flora and their associated wildlife (e.g. pollinators) in unpredicted ways. Damage produced by agricultural management of the agro-ecosystem, and indeed also in general environmental management, may overcome the costs of any environmentally-friendly weed control. Therefore, new weed management systems and assessment methods are necessary to evaluate the importance of weeds as virus reservoirs, even under low management regimes. From the standpoint of aphid control and associated virus transmission, the complete lack of any management needs to be seriously reconsidered, more especially the virus vectoring capacity of some aphid species like *A. solani*, which is highly polyphagous. Because of the unpredictable high local aphid density on invasive weeds, aphid migration onto several local crops (e.g. potato, considered as both a low-cost and low-input crop) may potentially cause — and indeed probably does often cause — unpredictable virus infections, including under low-input management regimes. From this we deduce that cultivation methods involving invasive weed and insect vector control need to be reconsidered, even when and if no other management approach is planned. To this end, further research is planned to quantitatively test viral infestation and detect virus spread via weed-aphid-crop plants.

Whilst this pilot present study is not definitive in that individual virus vectoring aphids were not directly tested, nevertheless it represents one of the first contributions to this particular topic area. As such, the study details the pioneering nature of this broad approach, one that shows considerable promise in attempts to understand the role of weed reservoirs in weed-crop aphid borne virus transmission, thereby paving the way for the next publications planned from our group in this fascinating area of applied research.

## Data Availability Statement

The raw data supporting the conclusions of this article will be made available by the authors, without undue reservation, to any qualified researcher.

## Ethics Statement

All applicable international, national, and/or institutional guidelines for the care and use of animals were followed. The manuscript does not contain any studies with human participants performed by any of the authors.

## Author Contributions

A-KS, AB, and JK perceived and designed the experiment. ÉV, AH, and ED performed the small RNA HTS. A-KS, JB, A-KS, and AB performed the data collections. ZG and ED performed the RT-PCR. AH performed the Northern blot analysis. A-KS, AB, and HL performed the data analyses, and wrote the manuscript.

## Conflict of Interest

The authors declare that the research was conducted in the absence of any commercial or financial relationships that could be construed as a potential conflict of interest.
