# Supplementary material for: Local Aphid Species Infestation on Invasive Weeds Affects Virus Infection of Nearest Crops Under Different Management Systems – A Preliminary Study
Source: Front Plant Sci. 2020 Jun 25;11:684. doi: 10.3389/fpls.2020.00684 (PMC7330602; doi:10.3389/fpls.2020.00684)
Supplement: Supplementary file 1 [file Data_Sheet_1.docx]

**Supplementary online materials**

**Tables**

**Table S1.** Fertilizer and pesticide input on crops under high intensity management (HIF) in the two study years.

| Crop |  | Treatments |
| --- | --- | --- |
| Potato | Fertilizer | N, P, K (15,15,15) 0.2 t/ha |
|  | Herbicide | Sencore (metribuzin70%) |
|  |  | Titus 25 DF (rimsulphuron) |
|  | Insecticide | Calypso (tiacloprid) |
|  | Fungicide | Banjo (fluazinam) |
|  |  | Ridomil Gold (mefenoxam, mankoceb) |
|  |  | Infinito (62.5 g/l fluopicolide +  625 g/l propamocarb clorhidrat) |
|  |  | Consento (375 g/l propamocarb clorhidrat +  75 g/l fenamidon) |
|  |  | Acrobat Mz (difenomorf, mankoceb) |
| Alfalfa | Fertilizer | N, P, K (15,15,15) 0.16 t/ha |
|  | Herbicide | Pallas (piroksulam) |
|  | Insecticide | Fastac (alfa-cipermetrin) |
|  |  | Falcon Pro (protioconazol 53 g/l +  spiro­xamină 224 g/l + tebuconazol 148 g/l) |
|  | Fungicide | Amistar Xtra (azoxistrobin) |
| Maize | Fertilizer | N, P, K-15,15,15 0.15 t/ha |
|  | Herbicide | Adengo (isoxaflutol 225 g/l +  tiencarbazon-metil 90 g/l +  ciprosulfamide (safener) 150 g/l) |

**Figure S1**. *E. annuss* in LIF and HIF, and the surrounding landscape.

**Table. S2.** Summary of the samples used for small RNA library preparation.

| **Place of sampling** | **Library ID** | **sampled plants** | **Date of sampling** | | |
| --- | --- | --- | --- | --- | --- |
|  |  |  | **2017** | | |
|  |  |  | **June** | **July** | **August** |
| **HIF** | 1_TC | *S. tuberosum* | 10 | 10 | 10 |
|  |  | *M. sativa* | 10 | 10 | 10 |
|  |  | *Z. mays* | 10 | 10 | 10 |
|  | 2_TB | *E. canadensis* | 10 | 10 | 10 |
|  | 3_TS | *E. anuus* | 10 | 10 | 10 |
| **LIF** | 4_SC | *S. tuberosum* | 10 | 10 | 10 |
|  |  | *M. sativa* | 10 | 10 | 10 |
|  |  | *Z. mays* | 10 | 10 | 10 |
|  | 5_SA | *S. canadensis* | 10 | 10 | 10 |
|  | 6_SS | *E. anuus* | 10 | 10 | 10 |

**Table S3.** Initial statistics of the sequenced small RNA libraries. Samples were sequenced using HiScan2000 by UD Genomed (Debrecen, Hungary) 50 bp, single end. Fastq files of the sequenced libraries were deposited to the GEO and can be accessed through series accession number GSE132755. Initial statistics were done using CLC Genomic workbench.

| **Site** | **Library code** | **Sequenced reads** | **Trimmed reads (containing redundants)** | **Non-redundant reads** | | **Number of contigs** |
| --- | --- | --- | --- | --- | --- | --- |
|  |  |  |  |  | **%** |  |
| HIF | 1_TC | 9 444 123 | 9 212 041 | 2 019 811 | 21.93 | 3553 |
|  | 2_TB | 16 287 256 | 15 202 422 | 3 423 785 | 22.52 | 4524 |
|  | 3_TS | 20 087 212 | 19 915 587 | 6 246 615 | 31.37 | 19 038 |
| LIF | 4_SC | 21 087 654 | 20 477 950 | 7 192 941 | 35.13 | 11 047 |
|  | 5_SA | 17 177 187 | 16 751 341 | 5 537 461 | 33.06 | 10 164 |
|  | 6_SS | 13 580 569 | 13 457 370 | 5 116 560 | 38.02 | 6 142 |

**Table S4.** Summarized result of the bioinformatics analysis of the sequenced libraries.

**Table S5.** Detailed result the bioinformatics analysis of the sequenced libraries using stringent parameters in details for viruses which presence were further studied by RT-PCR or by Northern blot.

**Table S6**. Sequence of the used of PCR primers for virus detection with their appropriate references.

| **Primer Name** | **Primer Sequence (5'-3')** | **Position on the reference genome** | **Genome used as a reference** |
| --- | --- | --- | --- |
| **PVY CP_8503F** | AAGGATCCGCTTTCACTGAAATGATGG | 8503-8530 | NC_001616.1 |
| **PVY CP_9701R** | AGGGAAGCTTCTAGAGTCTCCTGATTGAAG | 9701-9671 |  |
| **PVS-1793F** | CATTCCAGGCATAACGTTAA | 1793-1812 | NC_007289.1 |
| **PVS-2814R** | TTCATATCTTGCACGCTCAC | 2814-2795 |  |
| **PVM_7225f** | ATGGGAGATTCAACGAAGAA | 7225-7244 | NC_001361.2 |
| **PVM_8462r** | CTACTCTCGCTTGTTGATGAC | 8462-8442 |  |
| **PVX_5927_S** | GGACATGAAGGTGCCCAC | 5927-5945 | NC_011620.1 |
| **PVX_6475_AS** | GAAACTGGGGTAGGCGTC | 6475-6457 |  |

**
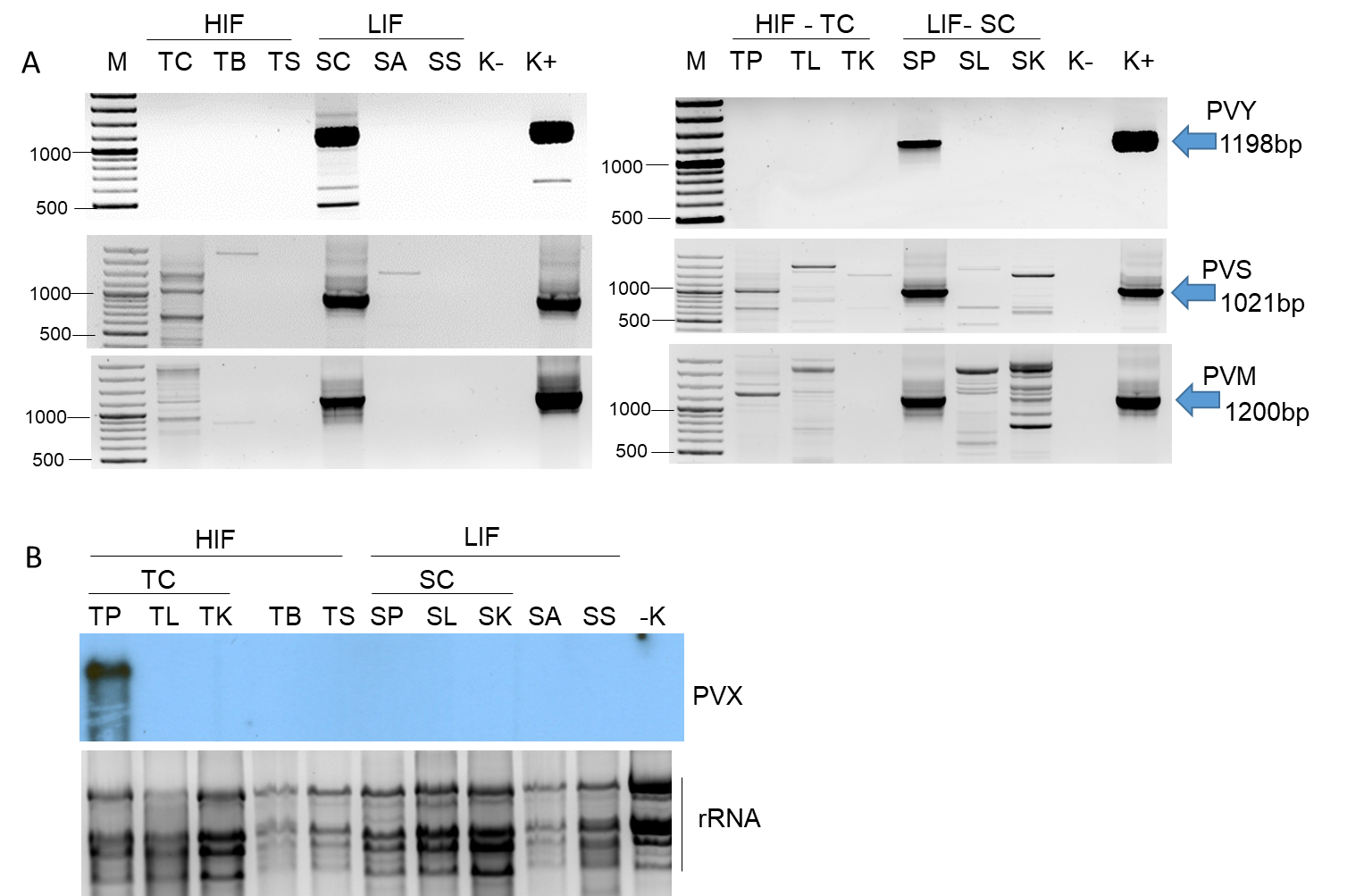
**

**Figure S2.** RT-PCR validation of sRNA HTS using A/ RT-PCR or B/ Northern blot. cDNA was synthetized from RNA extracts representing each library or different crops in the library using random primers and used as templates for PCR reactions with diagnostic primers (Stable4). PCR products were analysed by agarose gel electrophoresis. (M): GenRuler 100bp+; (+K): cDNA containing the tested virus was used as positive (K+), or (K‒), with water as the negative control. For Northern blot, 4µg total RNA from pooled samples was separated on 1.2% agarose gels, blotted to Nytran membrane and hybridized with radioactively labelled PVX virus specific probes. Relative gel loadings are indicated by ethidium bromide staining of ribosomal RNAs.
